# Cancer cell response to extrinsic and intrinsic mechanical cue: opportunities for tumor apoptosis strategies

**DOI:** 10.1093/rb/rbae016

**Published:** 2024-02-20

**Authors:** Jun Shu, Huan Deng, Yu Zhang, Fang Wu, Jing He

**Affiliations:** National Engineering Research Center for Biomaterials, College of Biomedical Engineering, Sichuan University, Chengdu 610064, PR China; National Engineering Research Center for Biomaterials, College of Biomedical Engineering, Sichuan University, Chengdu 610064, PR China; College of Food and Biological Engineering, Chengdu University, Chengdu 610106, PR China; National Engineering Research Center for Biomaterials, College of Biomedical Engineering, Sichuan University, Chengdu 610064, PR China; National Engineering Research Center for Biomaterials, College of Biomedical Engineering, Sichuan University, Chengdu 610064, PR China

**Keywords:** mechanotransduction, cancer cell, apoptosis, mechanical stimulation, anticancer materials

## Abstract

Increasing studies have revealed the importance of mechanical cues in tumor progression, invasiveness and drug resistance. During malignant transformation, changes manifest in either the mechanical properties of the tissue or the cellular ability to sense and respond to mechanical signals. The major focus of the review is the subtle correlation between mechanical cues and apoptosis in tumor cells from a mechanobiology perspective. To begin, we focus on the intracellular force, examining the mechanical properties of the cell interior, and outlining the role that the cytoskeleton and intracellular organelle-mediated intracellular forces play in tumor cell apoptosis. This article also elucidates the mechanisms by which extracellular forces guide tumor cell mechanosensing, ultimately triggering the activation of the mechanotransduction pathway and impacting tumor cell apoptosis. Finally, a comprehensive examination of the present status of the design and development of anti-cancer materials targeting mechanotransduction is presented, emphasizing the underlying design principles. Furthermore, the article underscores the need to address several unresolved inquiries to enhance our comprehension of cancer therapeutics that target mechanotransduction.

## Graphical Abstract

**Figure:**
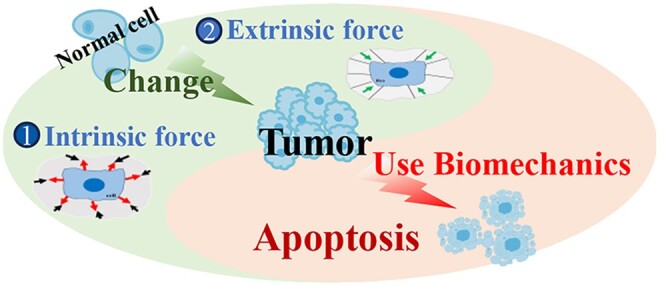

## Introduction

Cells are highly sensitive to mechanical signals from the extracellular microenvironment, as to chemical signals. Cells can maintain their mechanical stability through tensegrity structures [1]. The linker of nucleoskeleton and cytoskeleton (LINC) complex-cytoskeleton-focal adhesion-extracellular matrix (ECM) connection facilitates the transmission of extracellular mechanical information, including external stresses such as shear stress, circumferential stretch, ECM ligation and interstitial pressure, as well as intrinsic stresses resulting from cellular tractions through adhesions with neighboring cells and the ECM. This transmission occurs through mechano-chemical transduction, ultimately influencing cell activation, transcription regulation and metabolism. During this process, effector molecules, such as Yes-associated protein (YAP) and tafazzin protein (TAZ), may become activated, rendering cells more adaptive to survival in complex mechanical environments or expediting cell death [2, 3].

**Figure 1.**
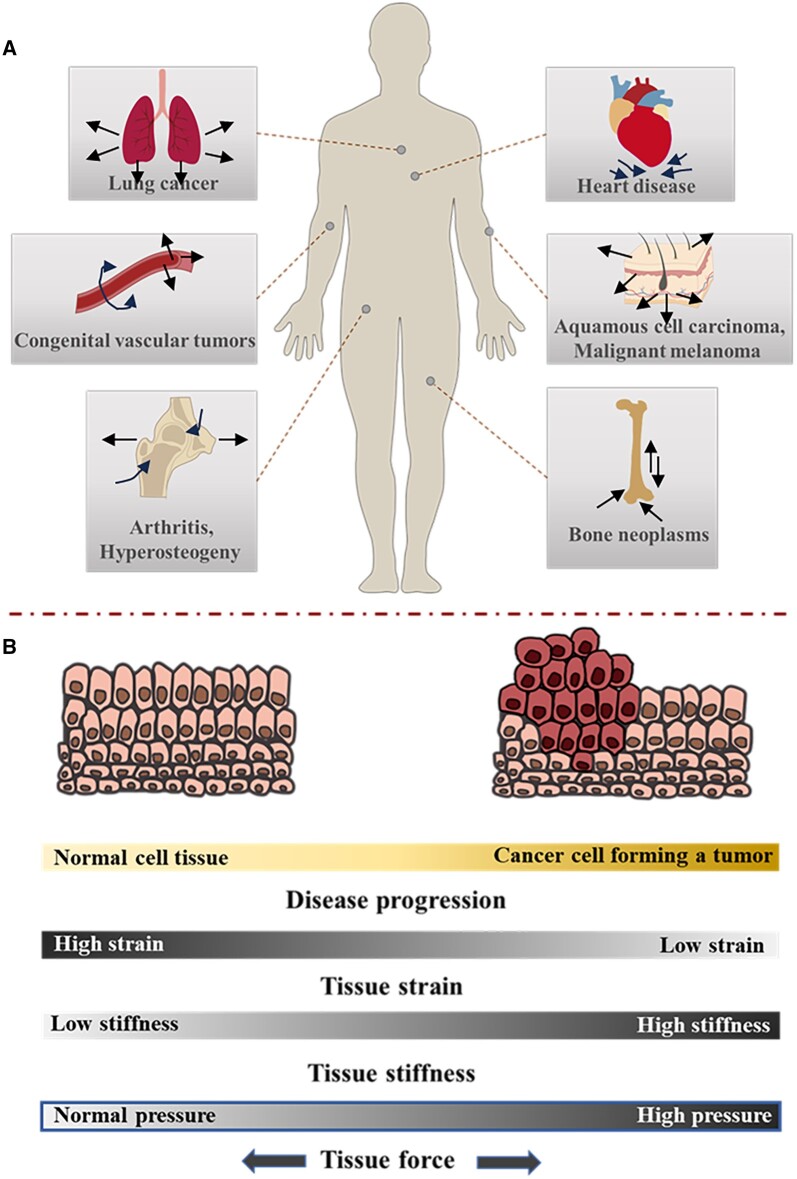
Mechanical stimuli in mechano-associated cancer and diseases in the human body. Cells in the different tissues of the human body experience mechanical stimuli and deformation. (**A**) There are examples of common forces, stresses, loads, and deformations that are shown, as well as possible mechano-associated diseases. (**B**) The conversion of a normal cell to a cancer cell is frequent along with alterations in mechanical properties.

How cells sense and respond to the mechanical signals of the extracellular microenvironment are closely related to the occurrence and development of various cancers and diseases [2] (Figure 1). The viscoelasticity of cells is expected to undergo adaptive alterations in response to the mechanical properties of the ECM. For instance, non-malignant mammary epithelial cells grown in a 3D laminin ECM show their viscoelasticity similar to that of the local ECM, while breast cancer cells cultured in these environments are more rigid [4]. Several changes occur in the microenvironment during tumor development, including the significant increases in matrix stiffness, interstitial flow shear force, interstitial fluid pressure and solid stress of tumor growth. These mechanical changes are mutually associated with the occurrence and progression of cancer [5]. Meanwhile, the change in the tumor microenvironment affects the behavior of normal cells around the tumor, such as fibroblasts and macrophages, further reshaping the ECM of tumor cells and promoting tumor growth and invasion.

Tumor development can be counteracted by the stimulation of apoptosis or other programmed cell death [6–8]. Considering that cancer is marked by uncontrolled and uncoordinated cell proliferation and survival, implementing strategies to inhibit cell proliferation and promote apoptotic cell death within tumors has proven effective in cancer treatment [9, 10]. Recently, it has been recognized that physical forces such as stretch, strain and tension play an important role in regulating the process of cell apoptosis.

**Figure 2.**
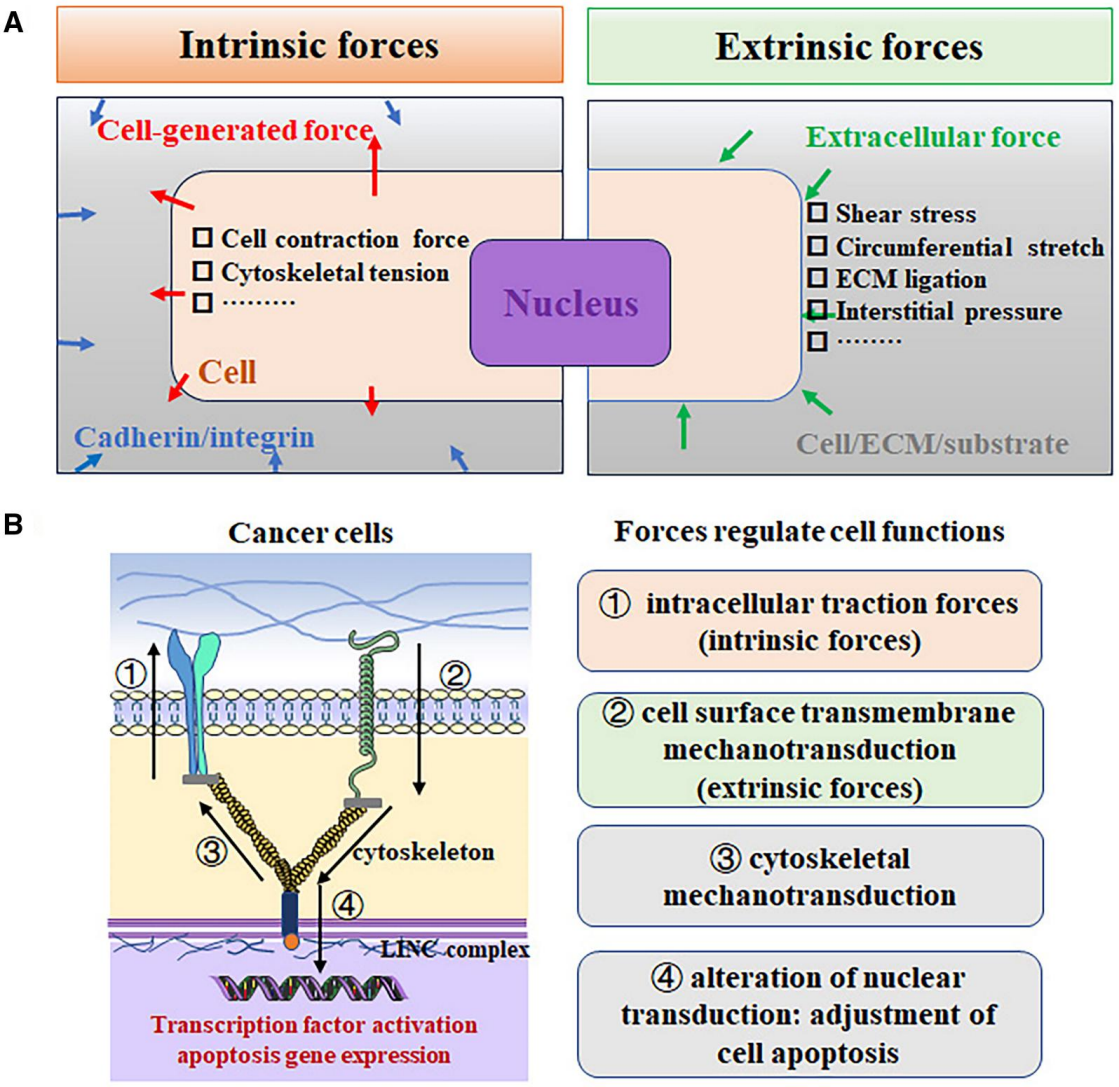
Cancer cells exert forces and are subject to external forces, which modulate their intracellular signaling pathway and thereby influence their apoptosis. (**A**) Within cells, intrinsic or cell-generated forces are generated and transmitted to other cells through cell–cell junctions, like cadherin receptors or through traction on adhesion ligands bound to integrins in the ECM. Extrinsic forces are the external forces applied to cells by shear or tension and/or compression. (**B**) Force is applied to cancer cell mechanotransduction mechanisms allowing them to detect physical cues from their microenvironment and to transduce and biochemically amplify these signals to regulate cancer cell apoptosis.

The mechanical cues to which cells are subjected can be broadly divided into two categories (Figure 2). One class, extrinsic forces are from blood flow-induced shear stress and circumferential stretch, externally applied mechanical strain, fluid flow-induced shear stress and ECM ligation [11–15]. Another class, intrinsic forces are from applied cellular traction through cell–cell and cell–ECM adhesions, as well as cell migration. The endogenous machinery of the cell, including tensile (actin) and compressive (microtubule) elements, and FA attachment complexes, participate in intracellular signaling [16]. Through the regulation of intracellular contractile forces and anchoring forces to the underlying substrate, cells achieve the preservation of a nuanced mechanical equilibrium [17]. This intricate and dynamic balance is governed by structures of cell-substrate adhesion, cytoskeletal configurations and the spatiotemporally controlled magnitude and distribution of internal contractile forces [18, 19]. Moreover, the interplay between the dynamic equilibrium and disequilibrium of forces exerts a significant impact on tissue functionality, regeneration, angiogenesis and the metastasis of cancer. This phenomenon holds paramount importance in the advancement of research and treatment methodologies for patients afflicted with various ailments, including cardiovascular disease, cancer and aging. Furthermore, it serves as a driving force for progress in the realms of regenerative medicine and the production of artificial organs [20, 21].

In prior studies, mechanical signals have been identified as critical regulators of cell behavior, tissue regeneration, and disease occurrence and treatment [22–24]. This study delves into the fundamental mechanism of mechanical signal transduction and its influence on tumor cell behavior, exploring three aspects: extrinsic force, intrinsic force and the effect of forces on tumor therapy, mainly focusing on cancer cell apoptosis. Through this review, we aim to provide readers with a deeper understanding of mechanics and their potential applications in tumor therapy.

## Intrinsic force-mediated tumor cell apoptosis

### Intrinsic force generated from cell–cell and cell–ECM adhesion and migration

In response to the local stiffness of 2D substrates or 3D microenvironments, cells regulate their force generation by pulling on the ECM, i.e. ‘inside-out’ mechanical stimulation. These processes depend on ECM adhesions, which transmit force between the ECM and the cellular cytoskeleton to transmit force, with myosin-based contractility serving as an important regulator of cellular traction (contractile) forces [25]. Indeed, these intracellular forces, including cell contraction force and cytoskeletal tension, play a regulatory role in signaling pathways involving in fundamental aspects of cell, such as cellular transformation to neoplasm, tumor formation and progression.

Contractile force refers to the force generated by cells in a 3D environment, which is resisted by the mechanical properties of the microenvironment. During the process of cellular migration, it actively engages in various cellular events, including adhesion with the substrate, formation of leading-edge protrusions, generation of cell body protrusions, translocation of the cell body and establishment of cell polarity. For example, cells need to exert forces against the substrate to move forward [26]. In migration, the cell pulls continuously on the matrix and neighboring cells, causing the contractile forces to be dynamically generated by the action of myosin II on the cytoskeleton, thus providing a lasting mechanical stimulus [27].

Cellular tension is governed by the cytoskeletal structure, influencing both cell shape and mechanical load bearing. Subcellular organelles are influenced by the forces within this tensed actin cytoskeleton, the stiffest and largest of which is the nucleus. Bonse *et al.* [28] demonstrated that cytoskeletal tension could regulate YAP nuclear localization, especially under pathological condition . YAP nuclear accumulation and transcriptional activity are enhanced by high cytoskeletal tension in cancer-associated myofibroblasts [29].

In order to transmit force to the ECM, a functional force linkage is formed between intracellular, contractile, force-generating motor proteins, the cytoskeleton and transcellular adhesions (e.g. integrins). As well as being closely linked to the actin cytoskeleton, cadherin-mediated cell contacts between neighboring cells are also subject to and respond to cell-to-cell forces. In the context of cancer, the disruption of tensional homeostasis can dysregulate integrin expression and activation, focal adhesion protein assembly, cytoskeletal structure and cell-to-cell and cell-to-ECM adhesions, thereby promoting cancerization.

### Intrinsic cytoskeleton force

Internal forces are generated by the cytoskeleton and then transmitted to the nucleus by LINC complexes or to other cells and the ECM through cytoskeleton-transmembrane receptors. The cytoskeleton is a network of biopolymers, including actin filaments, microtubules, intermediate filaments, motility proteins, cross-linked proteins, actomyosin complexes and regulatory proteins, within living cells that confers mechanical structure to cell, and transmits physical forces to and from the ECM surrounding them [30]. The actin filaments, microtubules and intermediate filaments comprise the main components of cellular rigidity [31]. Interference with the synthesis and assembly of actin, myosin or microtubule can severely weaken the tension generated by the cell, affecting cell spread and migration [32]. Actin filaments are closely bound to all components of the cell, forming a huge network connecting the nucleus, various organelles, and cell membrane, which are the largest contributor to the elastic modulus of the cell [31, 33]. A well-developed actin cytoskeleton produces a higher tension level, which gives the cells a wider adhesion to the substrate and a higher resistance to shear deformation [34]. It is becoming clear that the cytoskeleton plays an important role in mechanical force transduction to the cell interior [35–37].

The reciprocity between cancer cells and their intrinsic mechanical surroundings functions as a feedback system, empowering cancer with considerable adaptability across progressive stages. At the core of this feedback system lies the mechanical program involving F-actin and mechanoresponsive proteins, such as non-muscle myosin II, actinin, filamin and potentially others [38]. Studies indicate minimal changes in the mechanical properties of cells upon carcinogenesis, with preserved regular arrangements of cytoskeletal structures [39]. Nevertheless, heightened migration and invasion potential in cancer cells lead to pronounced changes in the expression of this mechanical network, facilitating significant spatial and temporal reorganization of the cytoskeleton in metastatic cells. Unsurprisingly, diverse protein levels of critical mechanobiome components and the broader actin cytoskeleton have been observed across various cancers. In addition, major cancer drivers and signaling proteins exhibit altered expression patterns, influencing cell mechanics. YAP, overexpressed in numerous cancers, modulates cellular actin architecture, nonmuscle myosin II regulatory light chain expression and phosphorylation, thereby impacting mechanical parameters, notably cortical tension, and deformability. Early activating KRAS mutations, prevalent in over 90% of pancreatic cancers and at high rates in colorectal and lung cancers, result in increased deformability and altered contractility [40]. Tumor cells with a high metastatic propensity manifest augmented softness, deformability and heightened intracellular molecular mobility, leading to increased diffusion [41–43]. This phenomenon is attributed to the necessity for cells to disseminate from the primary tumor and undergo intravasation to the systemic circulation, prompting the remodeling of their actomyosin cytoskeleton and increased deformability [44]. Tumor cells are frequently softer than normal cells, suggesting an adaptive softening during invasion and metastasizing [45, 46]. RAS/MAPK signaling pathway and EGFR signaling pathway mediate cytoskeletal remodeling and increase cell hardness. Restoring the mechanical properties of cancer cells by blocking these signaling pathways emerges as a reliable strategy to inhibit metastasis and spread of cancer cells [45, 47].

The alteration of cytoskeleton-mediated cellular forces plays an important role in the process of cell apoptosis. The manipulation of actin cytoskeleton recombination, achieved through the expression of Maspin protein in tumor cells, leads to the manifestation of epithelioid morphology in these cells. Additionally, this manipulation results in reduced cell migration, enhanced intercellular adhesion, and increased occurrence of apoptosis [48]. The relationship between cell structure, cellular force, and the expressions of apoptotic proteins is closely intertwined. For example, following exposure to carbon ion irradiation, the cancer cell cytoskeleton undergoes gradual disintegration, resulting in a decrease in cell hardness and volume, along with the formation of apoptotic bodies. Meantime, the expression of caspase-3 is negatively correlated with the increase of cell hardness, and the ratio of Bax/Bcl-2 is also gradually increased [36]. Studies have shown that elevated temperature (40°C) disrupts mechanical equilibrium in tumor cells, resulting in G1 phase arrest, senescence and apoptosis. This effect is attributed to the downregulation of F-actin expression and its associated generation of cell traction [49]. Besides, changes in cell mechanics prompt a more rapid apoptosis of tumor cells compared to biological signals [50]. Before the activation of the death receptor CD95/Fas, cytorelaxin B induces initial filamentous actin depolymerization, leading to decreased cell hardness, volume and intracellular molecular movement space. This process results in increased membrane prominence and roughness, ultimately culminating in the apoptosis of HeLa cells [50]. In addition, cytoskeletal deformation and recombination also play important roles in cell division and lysis [51, 52]. When external forces are excessive to the point where cytoskeletal remodeling and deformation cannot be fully offset, or when they remain unrelieved for an extended period of time, other cellular structures may become damaged, which would eventually lead to cell apoptosis or necrosis [53].

### Nuclear mechanics and nuclear mechanotransduction

**Figure 3.**
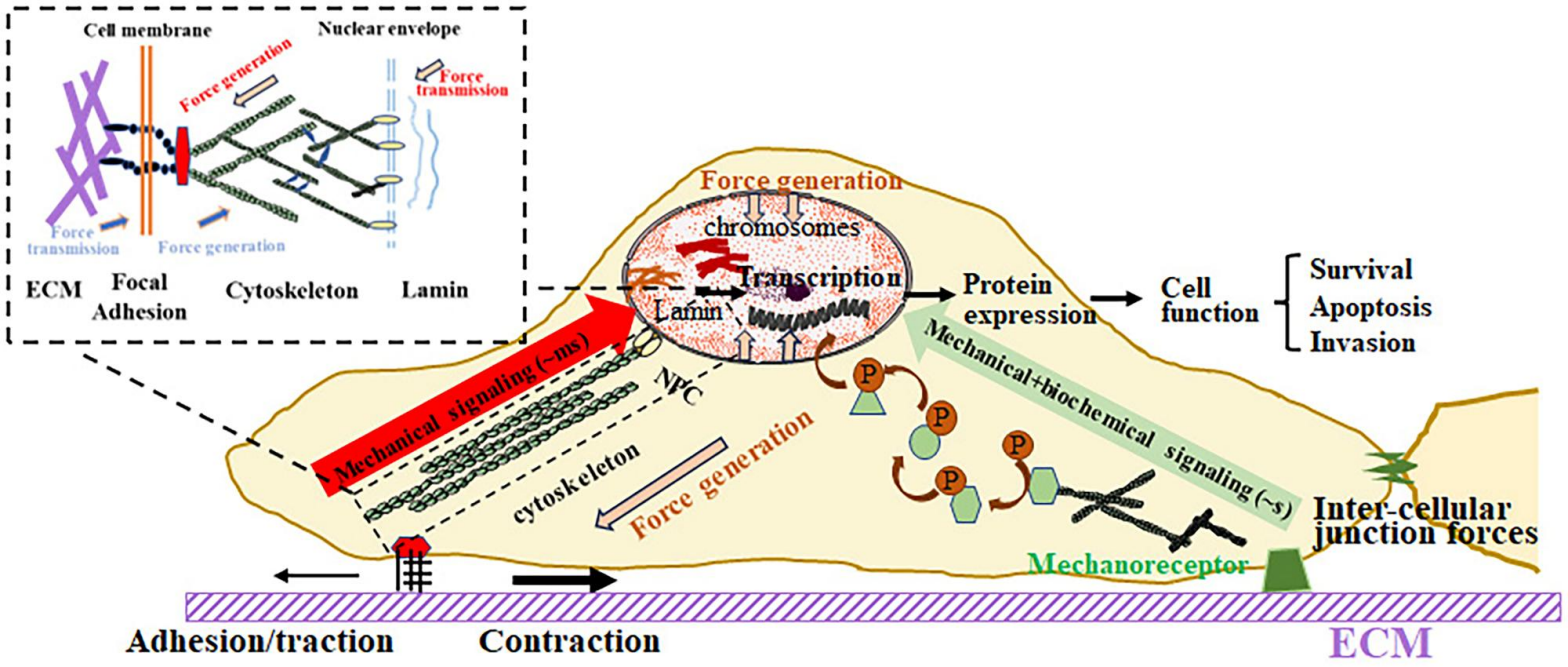
The extracellular and cytoplasmic force transduction by direct FA-cytoskeleton-LINC and indirect signaling cascades.

Commonly regarded as the cellular ‘brain’, the nucleus governs the comprehensive mechanical response of the cell. There are roughly two parts to the nucleus, the nuclear envelope and the nuclear interior. Typically, it is 2–10 times stiffer than the surrounding cytoplasm and exhibits both elastic (the nuclear lamina and nuclear envelope) and viscoelastic (the nuclear interior) behaviors [54–56]. In recent years, several studies have documented alterations in nuclear envelope composition in cancer. Denais and Lammerding [57] have reported the pivotal role of the nuclear envelope in cellular mechanics and function, encompassing the regulation of nuclear deformability, fragility and involvement in mechanotransduction signaling. There are two main ways in which extracellular and cytoplasmic forces are transmitted across the nuclear envelope to the nuclear interior: direct FA-cytoskeleton-LINC and indirect signaling cascades (Figure 3) [58].

Direct force transduction serves as a rapid and efficient mechanism transmitted to the nucleus at a considerably higher speed compared to chemical signals and molecular motors. This transmission occurs through a high-speed network established by the physical coupling of FA, cytoskeleton and nuclear membrane complex [59, 60]. Upon cellular stretching, tensile force is directly conveyed to the nucleus through stress fibers, which leads to nuclear deformation and the generation of non-uniform stiffness, viscoelasticity and strain within the perinuclear region and the nucleus [61–64]. Upon reaching a certain threshold of extracellular shear force or compression stress, chromatin movement is enhanced through the actin network, which causes alterations or depolymerization of chromatin conformation, ultimately promoting specific gene expressions [65–69]. Alternatively, evidence suggests that Lamin type A, a major contributor to nuclear mechanics, is physically connected to the perinuclear actin cap via the LINC (the link between the nuclear skeleton and cytoskeleton) complex, further transmitting cytoskeletal forces to the nucleus, inducing chromatin remodeling, transcription activation and gene expression with important implications in cancer progression [70, 71]. Cancer cells exhibit abnormal nuclear morphologies, such as invaginations, irregularities in volume and shape, as well as aberrant chromatin regions, in contrast to normal cells. These morphological variations contribute to alterations in the mechanical properties of cancer cell nuclei. For example, abnormal nuclear mechanics in MDA-MB-231 breast cancer cells lead to “cap” bumps in the cell and nucleus. This could be inhibited by Lamin A/C overexpression [72]. The overexpression or silencing of Lamin A would effectively regulate multiple oncogenic processes, including proliferation, apoptosis, embryonic development, tumorigenesis, epithelial-to-mesenchymal transition (EMT) and metastasis [73]. Meanwhile, overexpression of Lamin B1 inhibits the connection between the nuclear membrane and actin filaments, promoting the migration of melanoma cells [74].

Moreover, the nucleus can indirectly sense extracellular and cytoplasmic forces through signaling cascades. Various studies have demonstrated the presence of mechanical receptors on the surface of cell membrane. Following mechanical stimulation, post-translational modification or configuration changes open the cellular cascade signal network, facilitating the transmission of mechanical signals to regulate gene transcription in the nucleus [75]. Among them, the exploration and discovery of the new mechanical receptor and nuclear stress signal transduction mechanism have become a research hotspot. For example, Plexin D1, a member of the semaphorin family of stress receptors associated with both shear stress and tensile strain on the surface of vascular endothelial cells, forms a complex with the transmembrane glycoprotein neuropilin-1 and vascular endothelial growth factor (VEGF) receptor type 2, thus leading to the activation of the AKT and ERK signaling pathways [76].

Overall, the process of mechanotransduction does not operate in ‘one-way street’ and signals from the nucleus can be routed back to the cytoskeleton to change the way a cell perceives mechanical stimuli, creating a feedback loop in transcription. A deeper understanding of how nuclear mechanics and tumor progression interact may have profound implications for cancer diagnostics and treatment, and reveal new therapeutic targets for pharmacologically inhibiting cancer cell invasion.

### Cell membrane mechanics

The cell membrane is the most important part of the biofilm system, which is the barrier that keeps the cell isolated from the outside world. The cell membrane can be involved in the effects of cell mechanics on the cell behavior and function through the changes in membrane hardness, membrane tension, various force receptors and force-sensitive ion channels on the membrane surface. Lipid rafts are important structures in cell membranes that mediate cellular mechanics to regulate cancer metastasis and progression. They are rich in cholesterol and mechanically sensitive membrane proteins (such as Rho-type GTPase and integrin) [77]. The decrease in lipid rafts and the fatty acid chain length of phospholipid molecules cause a decrease in the stiffness of the cancer cell membrane, thus promoting the aggressiveness of cancer cells [78, 79]. Conversely, the increased stiffness of cell membranes can inhibit the invasion of cancer cells [79].

Membrane tension is the main factor regulating membrane deformation. Changes in membrane tension can be rapidly transmitted to the nucleus and various organelles, affecting the genetic and cellular events that can accelerate or inhibit cancer induction expression, movement, endocytosis and cytoskeletal remodeling [80–82]. The decreased membrane tension can improve the ability of membrane deformation, which is in favor of the metastasis of ovarian cancer cells to a certain extent [83]. Despite its functional significance, the membrane itself is relatively fragile. Tension in the plasma membrane primarily arises from attachment to the underlying actin cortex and osmotic pressure. The membrane is supported by the cell cortex, an F-actin scaffolding. The cell cortex is a thin shell of actin that lies underneath the plasma membrane and is part of the cytoskeleton. In mammalian cells, this cortex is approximately 1 μm thick and connects to the plasma membrane through specific protein links, as well as active proteins polymerizing actin locally. The cortex is a substrate for myosin attachment and tension buildup. Many of the ‘mechanosensitive elements’ in the membrane can be mechanosensitive because they are a part of the membrane-cortex complex, and in many cases, directly linked to the underlying cytoskeletal machinery and/or the ECM. For instance, the classical model of Piezo1’s physiological function posits its diffusion in the plasma membrane, locally activated by membrane bilayer tension or curvature changes resulting from cytoskeletal-dependent mechanical events [84].

Cell membrane itself is also a force sensor [85]. When cells are exposed to shear or tensile stress, the lipid order, fluidity, and cholesterol content of the plasma membrane would produce specific changes and guide cell behavior [86]. This may be related to the thinning of lipid bilayer in plasma membrane under stress, which leads to the exposure of force-sensitive channels or proteins such as TRPC6 [87].

Taken together, living cells, as complex soft materials, exhibit intriguing mechanical properties. An inherent mechanical program empowers cancer cells to continually sense and adapt to their environment, influencing tumorigenesis and development. The interactions between the cytoskeleton and membranes extend beyond mere structural or mechanical linkages, evolving into dynamic interactions that encompass newly identified functions related to signal transduction pathways. Exploring the mechanical system as a therapeutic target holds promise for more effective treatments in the future.

## Extrinsic force-mediated tumor cell apoptosis

**Figure 4.**
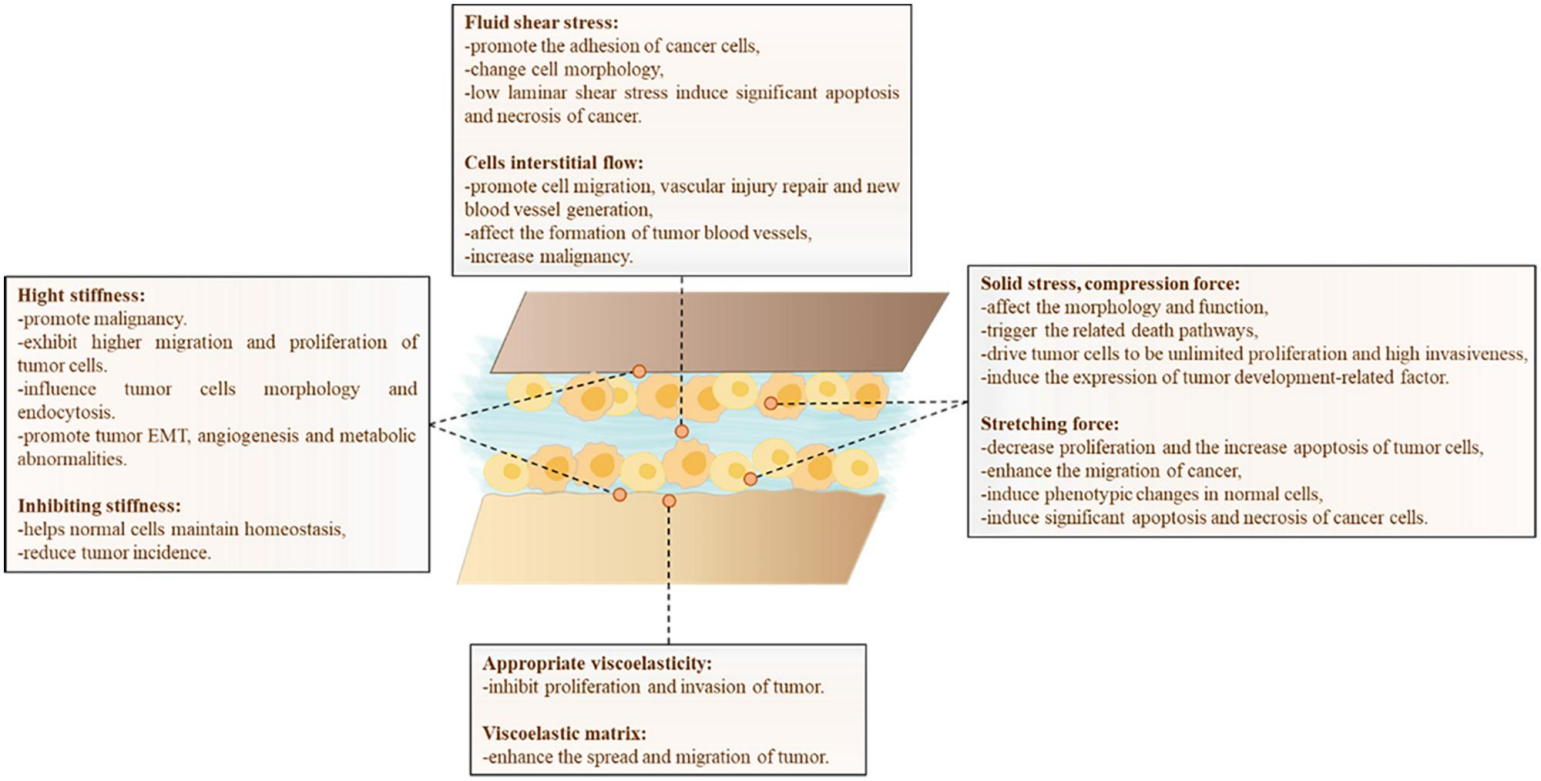
The extrinsic forces modulate the development and progression of tumors, including elasticity and viscoelasticity, shear force, interstitial flow, solid stress, compression force and stretching force.

Extrinsic forces, such as elasticity and viscoelasticity of ECM, shear force and pressure of fluid, compression force and tension force of tissue, directly or indirectly participate in the regulation of cell life activities. It has been demonstrated that mechanical stimuli, in addition to disrupting cellular homeostasis, establish significant connections with cancer [88, 89]. Cells are compelled to respond to these stimuli, either to preserve their integrity or initiate an appropriate response (Figure 4).

### Mechanical stresses generated from cells surrounding the extracellular matrix

The mechanical stress generated from cell surrounding the ECM is essential for cell adhesion, cell function and tissue development. The close connection between cells and their surrounding ECM allows them to keenly sense changes in their external environment. Abnormal metabolism of the ECM leads to changes in its structure and mechanical properties of ECM, contributing to the onset of various diseases, including tissue fibrosis, cancer cell proliferation, migration, EMT and so on [90, 91]. Therefore, the mechanical microenvironment of cells plays an important role in the generation, migration and progression of cancer. An ECM extends the cytoskeleton through integrins and convergent proteins, establishing a physical link between the ECM and the microfilaments of the cell. Studying the mechanical stresses between cells and their surrounding ECM can offer valuable insights into cancer development and aid in enhancing treatment options.

#### Matrix stiffness and elasticity

The stiffness observed in the ECM arises from the interactions between the ECM and cells, commonly understood as the mechanical forces exerted by the ECM on the cells. Deviations in stiffness can significantly impact various cellular processes such as differentiation, proliferation, tumor metastasis and drug resistance [92–94]. Tissue cells transmit forces to substrates as they exert pushing and pulling actions on their surroundings, facilitated by adhesion molecules like integrins and cadherins. Accordingly, the substrate’s resistance determines how well the cell can contract and migrate, causing the cell to adjust adhesion and reorganize cytoskeletal structures [64, 95–97]. It is complex mechanisms that allow cells to communicate and probe their microenvironment while also being sensitive to changes in substrate stiffness.

Each cell type specifically exists in the environment with a different modulus of elasticity or plays its function in the environment within a certain range of modulus of elasticity. For example, spinal cord neurons exhibit stellate spread at 50–550 Pa, myocytes exhibit myosin/actin stripes only at about 12 kPa of gel, and osteoblasts exhibit high cellular activity at 110 kPa [98]. The elasticity of the ECM determines the extent to which cells can influence their surroundings. The expression profile of ECM protein in tumor is significantly different from that in normal tissue. In response to increasing ECM elasticity, cells undergo a variety of changes at the molecular and cellular scales. Mammary epithelial cells exhibit loss of apicobasal polarity, enhanced focal adhesion assembly and increased proliferation [99]. Application of tensile forces to integrins triggers Rho signaling, leading to actin filament assembly, nuclear translocation of myocardin-related transcription factor A, and the expression of smooth muscle α-actin in fibroblasts [100]. ECM elasticity also induces shifts in protein localization, fostering malignant cellular behaviors. For instance, integrin clustering and activation on stiff matrices cause Rac1b to localize to the plasma membrane in mammary epithelial cells, resulting in upregulation of Snail and EMT [101]. YAP and transcriptional coactivator with PDZ-binding motif localize to the nucleus in mesenchymal stem cells cultured on stiff substrata, while they remain in the cytoplasm in cells cultured on the soft substrate [102]. Once in the nucleus, YAP and TAZ promote proliferation, and at elevated levels, this can result in neoplasia. Changes in the elastic modulus of the ECM are associated with the progression of many diseases, including cancer, fibrosis, atherosclerosis and so on (Table 1). The elasticity of tumor tissue is significantly higher than that of normal tissue. While type I collagen in normal tissue forms a loose network structure, collagen fibers in tumor tissue have a more consistent orientation. Historically, breast tumor macrohardness has been used to diagnose the disease and has also been strongly associated with local recurrence and metastasis [109].

**Table 1.** The mechanical properties of normal tissue and cancer tissue

	Normal tissue (kPa)	Cancer tissue (kPa)	Ref.
Breast	0.1–4	10–42	[103]
Brain	7.3 ± 3.6	∼35 (low-grade gliomas)	[104]
		∼50 (high-grade gliomas)	
Liver	<7.3	>17.5 (cirrhosis and fibrosis)	[105]
Prostate	16.0 ± 5.7	40.6 ± 15.9	[106]
Mammary gland	167 ± 31 Pa	4049 ± 938 Pa	[99]
Pancreas	<15	>40	[107]
Bladder	<5	5–15	[108]

Enhancing ECM stiffness promotes the transition from pre-malignancy to invasive malignancy, whereas inhibiting matrix stiffness reduces tumor incidence and improves cancer treatment effects. The increased stiffness of ECM causes the deeper folds at the nuclear membrane of breast cancer cells and alters the chromatin accessibility, which in turn promotes the malignant development of breast cancer through the Sp1-HDAC3/8 pathway [110]. ECM stiffness and laminin content control the expression of β-casein in normal mammary epithelial cells. The soft matrix helps cells maintain homeostasis, while stiff ECM and the loss of laminin signal promote the occurrence and development of breast cancer [111]. A similar phenomenon has been observed in glial cells within the human brain, where tumor glioma cells exhibit heightened migration and proliferation in response to increased matrix stiffness [112]. The increase of matrix elastic modulus in a 3D environment reduces the expression of normal hyaluronic acid membrane receptor CD44s and increases the mutant CD44v6, thereby promoting the EMT, angiogenesis and metabolic abnormalities of gastric cancer cells [113].

When examining the impact of ECM stiffness on various tumor cells, there are indeed some commonalities between different tumor cells. Specifically, tumor cells from different origins exhibit an enhanced migratory and invasive capacity with increased ECM stiffness, indicating a shared sensitivity to stiffness alterations and a more efficient adaptation to diverse tissue environments. Variations in ECM stiffness can activate common signaling pathways, including Rho GTPases and MAPK, influencing tumor cell survival, proliferation and migration. Activation of these pathways appears to be a shared response mechanism among tumor cells. Furthermore, alterations in ECM stiffness may collectively enhance adhesion and matrix interaction in tumor cells, representing a shared pathway for improved adaptation and execution of migration and invasion in stiffer environments. Despite these shared characteristics, notable distinctions exist among tumor cells. Consequently, a thorough examination of the collective response of tumor cells to ECM stiffness should appropriately acknowledge these diversities, fostering a more comprehensive comprehension of tumor cell behavior in mechanical environments.

Cytoskeleton-integrin pathway mediates the regulation of ECM stiffness on cell behavior. β4 integrin/Ras-related C3 botulinum toxin substrate 1(Rac1)/phosphoinositide 3-kinase (PI3K) is the pathway by which mammary epithelial cells sense matrix rigidity and adhesion. Normal mammary epithelial cells exhibit malignant phenotypes as a result of increased matrix stiffness, which inhibits the expression of α6β4 integrin in the semi-desmosomes. This malignant phenotype would disappear with the increase of ligands on the matrix [114]. In addition, within 3D matrices, prostate cancer cells exhibit a response to increased matrix stiffness characterized by a reduction in cell elasticity and an increase in intracellular viscoelasticity. This behavior may be linked to an increase in adhesion sites and integrin mediations in the 3D matrix [115]. Matrix stiffness also influences cell morphology and endocytosis, which is regulated by actin cytoskeletal recombinant proteins (phosphorylated cofilin, profilin, adhesion plaque kinase and vinculin) and intracellular proteins (Caveolin 1 and Rab 11) [116]. Adhesion plaque kinase (FAK) and PI3K are also involved in the regulation of matrix stiffness on cell behavior [117]. Notably, talin and actin cytoskeleton-integrin linking molecule can only grow to a sufficient length to bind to vinculin when the substrate stiffness is greater than 1 kPa, forming a stable actin-integrin connection, which can play a role in cell sensing ECM stiffness [118].

#### Matrix viscoelasticity

Biological tissues and ECMs are not purely elastic materials, like a rubber ball or a spring, since they exhibit complex, time- and rate-dependent mechanical behaviors, a property called viscoelasticity or poroelasticity [119, 120]. Viscoelastic materials have three common features: stress relaxation (constant strain resulting in time-dependent decreasing stress), creep (constant stress resulting in time-dependent decreasing strain) and hysteresis (the difference between loading and unloading processes) [121, 122]. Viscoelasticity as a mechanical property influences disease progression, together with or independently of stiffness. Matrix viscoelasticity appears to govern essential cellular processes, including behaviors not observed in cells cultured in purely elastic hydrogels, both in two- and three-dimensional culture surroundings [123]. Specifically, by considering the tumor ECM as a standard linear viscoelastic solid, there is evidence that an intermediate viscosity level can facilitate cancer cell spreading when the ECM stiffness is relatively weak, which mirrors the circumstance that the substrate relaxation time under such conditions is somewhere between the coupling bond time scale and its typical bond lifetime. In other words, viscosity serves to stiffen soft substrates, which encourages cell adhesion to the ECM and facilitates consequently cell spreading. As with high stiffness, the large stress carried by the couplings elicits an enhancement of their binding levels as well as an augmentation of integrin tightness (clutch amplification), thereby rendering the cell contribution to substrate stiffness to become a saturated response, and viscosity no longer to be an issue [124].

Malignant and benign cells have significant differences in their viscoelastic response [125, 126]. For instance, human hepatocellular carcinoma cells (Huh7) demonstrate enhanced spread and migration on viscoelastic substrates, while normal hepatocellular cells (PHH and LX-2) exhibit reduced spread and adhesion. This difference is related to the ineffective assembly of actin stress fibers in the viscoelastic matrix of normal hepatocytes [127, 128]. Additionally, this is accompanied by a shift in cellular morphology, as viscoelasticity induces an EMT phenotype. The migratory ability of MDA-MB-231 triple-negative breast cancer cells increases with a decreasing damping coefficient [129]. Appropriate viscoelasticity can also inhibit the proliferation and invasion of breast cancer cells by influencing normal bone cells to secrete more pro-inflammatory factors and chemokines [130]. Cell behaviors, including spreading, conformation, division and movement, are largely dependent on viscoelasticity, primarily determined by the supporting cytoskeletal systems [131]. Upon mechanical deformation, these mechanical loads from the microenvironment are transmitted to the nucleus via filament linkages, altering gene expression and consequently biochemical signaling as a result [132]. Therefore, the intricate interplay between cancer cells and the viscoelastic nature of microenvironment in which these cells reside is crucial for cellular mechanotransduction. This interplay guides cancer cell behavior and, in turn, prevents tumors from experiencing excessive growth and metastasis.

### Solid stress, compressive, tensile and shear

The rapid growth of tumors produces a large compression force on the cells inside the tumor, which can affect the morphology and function of cells and trigger the related death pathways [133, 134]. However, at the peripheral region of the tumor, the effect of compression force on cells is relatively small, promoting unlimited proliferation and high invasiveness of tumor cells [134]. The alterations in stress, interstitial fluid pressure and permeability in tumor are closely related to the compressible remodeling of ECM and collagen densification induced by excessive proliferation of cancer cells [135]. Rho/ROCK signaling pathway mediated F-actin activity and cyclin-dependent kinase inhibitor p21 are involved in the enhanced hardness of compressive stress-induced tumor spheroids, and the accumulation of compressive stress [136]. In a 2D environment, human fibrosarcoma cells sense compression force through Ca^2+^, TRPV4, Rho and YAP signaling pathways [137]. Meantime, intratumor compression forces also affect normal cells in and around the tumor. For example, intratumor compression forces would induce the expression of normal fibroblast growth differentiation factor-15 (GDF15) and ECM protein secretion in normal epithelial cells, as well as the activation of the β-catenin pathway, which further exacerbates tumor development [138, 139]. It is noteworthy that compression force promotes autophagy in HeLa cells via the lipid raft-mediated phosphorylation of p38MAPKs. Additionally, it enhances the invasiveness of HeLa cells by increasing paxlin turnover and MMP-2 secretion [140].

In addition, stretching force also plays an important role in the migration, apoptosis, and necrosis of cancer cells, and can play a tumor-killing role by enhancing the M1 polarization of macrophages. The stretching force promotes the expressions of Akt and caspase-3 in skin melanoma cells (B16F10) that are co-cultured with macrophages, leading to the decreased proliferation and the increased apoptosis of tumor cells. However, it has also been shown that stretching force enhances the migration of human ovarian epithelial cancer cells (SKOV-3). When cancer cells are exposed to periodic stretching, the cell would quickly respond via integrin α5β1 or ανβ3 independently mediated actin cytoskeletal remodeling [141]. The integrin α5β1 responds to stretching by adjusting the centripetal traction generated by long actin filaments, while integrin ανβ3 responds to stretching by adjusting short actin filaments to generate more flexible and dynamic random forces [141]. In ovarian cancer cells, actin also contributes to the response of G protein-coupled receptor 1 (OGR1) to acidification of the extracellular microenvironment under stretching conditions [142]. Simultaneously, stretching force induces normal tissue-associated fibroblasts (NAFS) to adopt a phenotype similar to that of cancer-associated fibroblasts (CAF) via the organization of the ECM fiber arrangement, which enhances the invasion and aggression of tumor cells [143]. Additionally, studies have shown that stretching can promote the spread and migration of breast cancer cells only in the initial cycle, while tumor cells would undergo significant apoptosis and necrosis after more stretching cycles [144]. Overall, reasonable control of stretching force may play a role in cancer treatment and prevention. Animal experiments have shown that stretching therapy in mice alone can effectively limit the growth of implanted tumors, with the stretching group showing a 52% smaller tumor volume than the non-stretching group [145].

### Fluid pressure

Human cells are usually immersed in a fluid environment. When the fluid flows, shear stress occurs. According to recent research, fluid shear stress also affects cancer cell proliferation, metastasis, adhesion and apoptosis [146]. For instance, the presence of elevated wall shear stress at the arterial bifurcation promotes the development of intracranial aneurysms [147]. Under the shear force of 13 dynes/cm^2^, endothelial cells secret more von Willebrand factor and thrombocytopresin-1 into ECM, which promotes the adhesion of breast cancer cells to ECM through integrin [148]. The incremental elevation of fluid shear stress within the microfluidic channel (ranging from 0.12–1.8 dynes/cm^2^) elicits a progressive transformation in the morphology of human glioma cells. This metamorphosis is characterized by an augmentation in cortical rigidity and nuclear softness. Concurrently, the cells exhibit a gradual shift in motility behavior from crawling to rolling, coupled with an observed increase in adhesion forces [149]. Conversely, it is observed that low laminar shear stress results in substantial apoptosis and necrosis of cancer cells [150, 151]. However, investigations have revealed variability in the apoptosis mechanisms among different types of tumor cells. Melanoma is associated with integrin avb3 and AKT, while breast cancer cells are associated with concave-1. In liver cancer cells, FAK and integrin are involved together [152]. An additional study indicates a close association between the apoptosis of tumor cells in blood circulation and the generation and buildup of superoxide in mitochondria induced by fluid shear stress. Notably, cancer cells with high metastatic potential exhibit the ability to transform these superoxides into hydrogen peroxide through increased expression of MnSOD. This conversion facilitates the migration and adhesion of tumor cells, presenting a contrasting effect on their behavior [153].

In addition to the shear stress caused by the flow of blood and lymph fluid, the extensive flow of small interstitial fluid between tissues can also produce shear force and thereby affect the life activities of tissue cells [154]. The shear stress generated by interstitial flow is typically modest, yet it proves adequate for detection by the glycocalyx (the proteoglycan/glycoprotein layer located on the cell surface). This sensing mechanism outside the cell membrane initiates intracellular mechanotransduction processes [155]. The interstitial fluid flow induces endothelial cells and fibroblasts to produce MMP-1 and VEGF, which promotes cell migration, vascular injury repair and new blood vessel generation [156, 157]. For tumor cells, interstitial flow can also work in concert with other physical properties of ECM, such as matrix hardness, to promote cell migration [158]. Interstitial flow can cause stromal fibers to be arranged parallel to the flow direction and the uneven concentration of chemokines secreted by tumor cells such as tumor angiogenic factor TAFs, thereby inducing cell migration along the flow direction of the interstitial, affecting the formation of tumor blood vessels, and increasing malignancy [159, 160]. In addition, the tumor tissue has a higher interstitial fluid flow (∼3 μm/s) than the normal tissue, and the interstitial fluid continuously flows out from the tumor [161, 162]. This would induce the localization of FA-related proteins near the upstream membrane of the cell such as β1 integrin, nestin and FAK, leading to adhesion activation, polarization and countercurrent migration to the upstream direction of the interstitial flow of the breast cancer cells [161]. Under shear forces, normal cells also assist tumor development. Macrophages differentiate toward M2-type macrophages via β1 integrin/Src/Akt/FAK signal cascades upon exposure to tumor interstitial flow, which releases a larger number of factors associated with the EMT, migration, and invasion of cancer cells [162]. Furthermore, in co-culture with breast cancer cells MDA-MB-435S, interstitial flow triggers fibroblast migration and the remodeling of collagen matrix. This process leads to increased matrix density surrounding the cells, consequently enhancing the invasion ability of the tumor cells [163]. In tumor spheres formed through the co-culture of normal mammary epithelial cells (MCF-10A) and breast cancer cells (MDA-MB-231), the down-regulation of the intercellular adhesion protein E-cadherin in normal epithelial cell occurs due to the collaborative influence of mesenchymal flow and tumor cells, which promotes the invasion of the tumor [164].

### Other

In addition to elasticity, viscoelasticity and shear stress, interstitial pressure, hydrostatic pressure and osmotic pressure also have important effects on cell behavior. The competitive growth of tumor cells induces high interstitial pressure within the tumor, accelerating the outflow of interstitial fluid from the tumor and concurrently slowing the inflow from the external environment [165, 166]. Under high interstitial pressure, the overexpression of caveolin-1 and CAVIN1 in glioblastoma (GBM) mediates the upregulation of EMT-related proteins (such as MMPs), resulting in the promotion of tumor cell invasion [167, 168]. Through the downregulation of RYBP leading to p53 degradation, high interstitial pressure can promote the occurrence and development of breast cancer [169]. Tumor interstitial fluid and its pressure can activate the expressions of CLIC4 and integrin α11β1 in fibroblasts, promoting collagen secretion and ECM hardening, thereby increasing the proliferation and invasion abilities of breast cancer cells [166, 169]. However, exposure to higher osmotic pressure leads to reduced cell proliferation, migration, and dispersion in metastatic cancer cells [170].

In addition, increased hydrostatic pressure can induce the stiffening of endothelial cell membranes under constant tension, thereby activating swelling-associated sensitive ion channels and signal transduction pathways [171]. The leukemia cell line K562 and normal leukocyte exhibit divergent responses to sudden increases or decreases in hydrostatic pressure. Specifically, the sodium and potassium channels in K562 cells are activated, leading to reversible volume deformation. In contrast, normal leukocytes do not undergo such changes [172]. Hydrostatic pressure below 80 mmHg can promote the proliferation of human mesangial cells, while hydrostatic pressure above 100 mmHg can induce apoptosis and ECM deposition of human mesangial cells [173]. Besides, high osmotic pressure can synergistically interact with the acidic environment of cancer, affecting the cross-presentation of tumor-specific antigens by dendritic cells to allow tumor cells to escape the recognition and attack of immune cells [174].

Overall, in the tumor microenvironment, mechanical factors are pivotal. Tumor growth and development coincide with alterations in these mechanical factors, such as stress from uncontrolled tumor proliferation, augmented stromal stiffness, elevated interstitial hydraulic pressure and intensified interstitial fluid flow. Simultaneously, changes in mechanical factors exert influence on the tumor microenvironment, as evidenced by residual stresses compressing blood and lymphatic vessels, resulting in functional impairment and consequent perturbation of the metabolic milieu within the tumor, leading to interstitial hypertension. Moreover, mechanical factors have implications for tumor treatment and the metastasis of tumor cells. Notably, the rapid proliferation of tumor cells may compress the interstitial stroma into a convoluted and dense network, posing challenges for drug delivery. Additionally, the flow of interstitial fluid can influence the directional aspects of cell metastasis.

Furthermore, organizations exhibit intricate mechanical landscapes, wherein extrinsic and intrinsic cues frequently interact and cannot be easily separated. Forces acting on cells and those imposed on the extracellular environment contribute to stresses and deformations. Consequently, gene regulatory circuits may be redirected, leading to abnormal reactivation of embryonic developmental programs, which can drive cells to transform and cancer to initiate, as well as cause cancer progression and metastasis later during the course of the disease.

## Biomechanics guide tumor therapy

**Figure 5.**
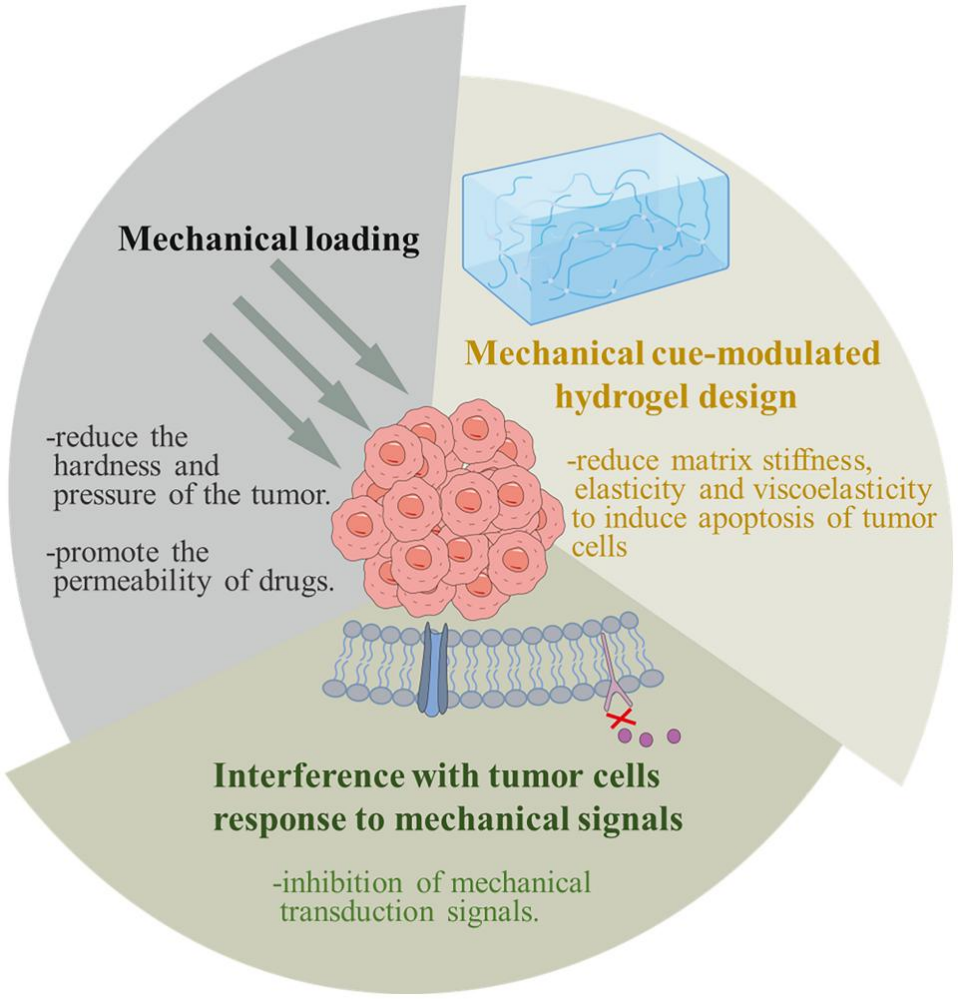
Biomechanics guide tumor therapy, including mechanical loading, mechanical cue-modulated hydrogel design and interference with tumor cell’s response to mechanical signals.

A potential application for the above finding lies in the tumor therapy. Tumor therapy and drug resistance are highly influenced by changes in mechanical properties of cell microenvironment. Tumor ECMs can protect tumor cells from apoptosis, either directly by physical restriction or indirectly by binding to cellular ECM receptors (such as integrins), thus forming resistance to chemotherapy drugs [175, 176]. Accordingly, modulating the mechanical properties of tumor ECM or blocking/affecting cell response to ECM mechanics may be an effective strategy to induce tumor cell apoptosis and overcome drug resistance (Figure 5).

### Mechanical cue-modulated hydrogel design

The multiscale biomechanics studies mentioned above offer valuable insights for the development of novel cancer therapies. The therapeutic strategy for tumor, especially solid tumor, has shifted toward targeting the tumor microenvironment, rather than the tumor itself, exerting stronger anti-tumor effects. In recent years, modulating the mechanical properties of the tumor microenvironment is a promising new therapeutic strategy in the treatment of cancer. Despite the limited research reports, it has promising clinical application prospects. Hydrogels can effectively simulate ECM, and their mechanical properties are easy to control, which has attracted extensive attention from scholars [177–182]. Currently, researchers are striving to manipulate the mechanical properties of hydrogels, including elasticity, viscoelasticity and so on, thus accelerating the apoptosis of tumor cells and inhibiting tumor progression.

Improved tumor cell apoptosis can be achieved by engineering appropriate mechanical microenvironments of tumor cells [183–186]. Reducing matrix elasticity to induce apoptosis of tumor cells is considered as an effective strategy. For example, Guo *et al.* [187] manipulate the mechanical strength of the matrix material. With the mechanical strength decreasing from 62 to 1.4 Pa, the apoptosis rate of ovarian cancer cells increases from 1.85% to 52.68%. Moreover, in the treatment of liver cancer, as the matrix stiffness gradually decreases from 500 to 100 kPa, caspase-3 and caspase-9 are activated in combination with the anticancer drug oxaliplatin, and the apoptosis rate increases from 20% to 35% [188]. While the significance of the mechanical microenvironment in tumor progression is gaining increased attention, understanding chemoradiotherapy resistance driven by mechanical microenvironment is still in its early stages. Further studies are required to elucidate the related mechanisms. For example, the establishment of hepatocellular carcinoma microspheres with varying microenvironmental stiffness is achieved by adjusting the mechanical strength of sodium alginate microspheres. The results indicate that, under the treatment of paclitaxel, cisplatin and 5-fluorouracil, respectively, the survival rate of hepatocellular carcinoma cells in the high stiffness environment (105 kPa) is significantly higher than that in the low stiffness environment (21 kPa) and the medium stiffness environment (70 kPa). This suggests that the high-stiffness environment significantly enhances chemotherapy resistance in liver cancer, possibly linked to stiffness-induced endoplasmic reticulum stress response [189]. In the context of cancer therapy, *in situ* gels represent a novel type of environmentally responsive hydrogel. These gels can serve not only as drug delivery carriers but also as providers of a mechanical microenvironment. Hence, through the integration of engineering and biology expertise, it becomes feasible to spatially control the stiffness microenvironment, potentially inducing tumor cell apoptosis and mitigating drug resistance in tumor cells.

Reproducing tissue elasticity and dissipative properties represents an emerging strategy in cancer treatment [190]. Most hydrogels, especially biopolymer-based hydrogels, exhibit both elastic and viscous properties. However, the progress in developing matrix materials capable of independently regulating various viscoelastic properties (such as viscosity damping coefficient, loss modulus, stress relaxation, loss angle, creep, viscous dissipation, etc.) has been slow for a considerable period, limiting our exploration of the effects of viscoelasticity on cells [191]. Recently, advances in materials processing techniques have been made in the development of viscoelastic matrix materials with good biocompatibility. Several dissipating processes may lead to the viscous properties of hydrogels, including the unbinding of weak bonds, polymer disentanglement, protein unfolding and molecule slipping [192, 193]. It has been demonstrated that the viscoelasticity of hydrogels can be modulated through polymer molecular weight or network chain length, crosslink type and density, hydrogel composition or concentration and degradation, and some combination of varying the above parameters [123, 194–197]. Recent studies have demonstrated that hydrogel viscoelasticity significantly affects cell spreading and apoptosis. For instance, Mooney *et al*. [198] conducted a study revealing that U2OS cells exhibit increased spreading when placed on soft substrates with stress relaxation, compared to cells on elastic substrates with an equivalent modulus. However, the spreading of these cells resembled that of cells on stiffer elastic substrates. Additionally, our latest findings observe variations in the apoptosis rate of human osteosarcoma MG63 cells under different viscoelastic stimulation conditions [199]. Therefore, it is important to take into account hydrogel viscoelasticity when engineering 3D mechanical microenvironment for tumor cells.

It is noteworthy that the roles of elasticity and viscoelasticity in regulating the biology of the various cell types within the tumor microenvironment-including fibroblasts, tissue-resident stem and differentiated cells, and immune cells cannot be ignored. This consideration is essential for the rational design of materials aimed at enhancing cancer cell apoptosis. In the design of biomaterials to achieve mechanical stability in regenerating or engineered tissue, it may also be necessary to decouple the local viscoelastic or elastic properties that cells sense from the larger, tissue-scale properties [200]. Therefore, the introduction of biomaterials with controlled mechanical properties, encompassing viscoelasticity or elasticity, may have a transformative effect on therapy.

### Mechanical loading

The dense ECM and high interstitial fluid pressure in the tumor limit the diffusion of drugs into the tumor sphere, which affects the therapeutic effect of chemotherapy drugs. By mechanical loading on the tumor, it may reduce the hardness and pressure of the tumor and improve the penetration efficiency of the drug, which exerts a direct killing effect on tumor cells. The combined treatment of unfocused ultrasound and microbubble can change the interstitial fluid pressure in rabbit VX2 tumor. When the ultrasound intensity reaches 3 or 5 MPa, the interstitial fluid pressure decreases steadily [201]. Fluid shear force can promote the permeability of doxorubicin to tumors, thereby reducing colorectal cancer spheroid survival [202]. Meantime, ultrasound induces significant apoptosis and necrosis of human histiocytic lymphoma cell (U937) in a non-lethal hypotonic pressure environment [203]. Furthermore, simultaneously reducing ECM hardness and intratumor pressure to promote drug penetration into the tumor is also an important idea of multi-target combination drug delivery [204–206].

### The perturbation in tumor cell response to mechanics

Interference with tumor cells response to mechanical signals is another way to regulate the mechanical microenvironment. Inhibition of mechanical transduction signals mediated by the specific membrane receptor, integrin family, FAK, Rho-associated kinase (ROCK) and YAP/TAZ may prevent cancer initiation and development, as well as reverse tumor drug resistance.

Cells can sense the mechanical information of ECM and regulate their own behavior by anchoring ECM with receptors on the surface of cell membrane [207]. For example, Tetraspanin CD82 is a membrane protein in mammary epithelial cell membranes that mediates adhesion and migration. The activation of CD82 inhibits the metastasis of breast cancer cells by regulating cell membrane tension through the litavin-1 and YAP pathways [208]. Therefore, designing some chemical ligands to bind to the force-sensitive receptors on the cell membrane to affect/hinder the interaction between tumor cell receptors and ECM would be an effective strategy to inhibit the development of cancer.

The additional efforts aim to target mechanotransduction pathways at their downstream components, such as Rho, ROCK and YAP/TAZ signaling (Table 2). Metastatic progression necessitates cancer cells to remodel their cytoskeletons, enabling them to cope with increased motility and interaction with ECM [216–219]. It is primarily accomplished through the activation of the RhoA main effectors ROCK [220]. There is evidence indicating a regulation of ROCK expression in several cancers, which is associated with a poor prognosis. Moreover, several studies have demonstrated that their activity contributes substantially to the progression of cancer [221–224]. Thus, ROCK is generally considered an important player in the progression and development of cancer, which results in considering the use of ROCK inhibitors in the treatment of cancers, such as small-molecule inhibitors against ROCK (Y27632 and Fasudil). Moreover, YAP and TAZ present attractive targets for cancer therapy, given that their elevated nuclear levels are associated with a broad range of aggressive cancers [225–227]. In addition, a direct inhibitor of YAP/TAZ, verteporfin, can suppress the communication between YAP and its binding transcription factor TEAD, therefore inhibiting YAP/TEAD downstream signaling in various cancers, including retinoblastoma, malignant pleural mesothelioma, endometrial cancer cells and so on [228–232]. Hence, the development of ROCK and YAP/TAZ inhibition would be the efficient strategy for cancer treatment, but the use of these inhibitors for anticancer purposes should be tightly controlled to avoid adverse effects.

**Table 2.** Effects of mechanotransduction-targeted drugs in cell treatment

Agent/material	Action mechanism	Cells	Outcomes	Mechanical cues	Ref.
Gsmtx4	Block CaN/NFAT1 signaling axis	Rat primary chondrocytes	Protect chondrocytes from apoptosis and anabolic/catabolic imbalance under mechanical strain	Inhibited the deleterious effects of mechanical strain	[209]
Andrographolide	Activating the MAPK/Nrf2/HO-1 signaling pathway	Nucleus pulposus cells	Inhibits static mechanical pressure-induced apoptosis and improves cell viability	Suppress static mechanical pressure-induced ROS accumulation in the NPCs	[210]
γ-Fe_2_O_3_ SPIONPs	The opening of the Piezol mechanosensor	Neural stem cells	Regulates the directional differentiation of NSCs and neuron regeneration	Increase the elastic modulus of the NSCs	[211]
Y-27632	Inhibit RhoA/ROCK pathway	Unknown	Reduce internal resistance	Stiffness-induced cell activation	[212]
Morin	Inhibit Hippo/YAP and TGF-β1/Smad pathways	Hepatic stellate cells (HSCs)	Show antifibrotic effect	Stiffness-induced HSC activation	[213]
Xanthohumol	Mediate the GAS5/miR-27a signaling pathway	Chondrocytes	Protected chondrocytes and increased viability	Protective effects against mechanical stimulation-induced ECM degradation	[214]
Metuzumab	Decrease β1 integrin/FAK/Akt activation via CD147 blockade	Hepatocellular carcinoma (HCC)	Inhibit tumor growth and metastatic potentials	Stiffness, shear stress or IFP-induced proliferation and metastasis of HCC cells	[215]

Taken together, tumor biophysics offers novel insights for an enhanced comprehension of cancer, playing a pivotal role in the exploration of innovative therapeutic approaches. The emerging domain of mechanics-based design in oncology therapeutics focuses on harnessing the mechanical attributes of the tumor microenvironment to optimize treatment effectiveness. This encompasses the modulation of matrix mechanical properties to influence tumor cell behavior, the development of mechanosensitive drug delivery systems and direct mechanical interventions on tumor tissue. The future trajectory of this field anticipates heightened individualization and precision, fostering breakthroughs in cancer treatment to elevate therapeutic efficacy while mitigating adverse effects.

## Challenges and future perspectives

Major challenges persist in understanding the mechanical characteristics of the tumor microenvironment and the concept of mechanomedicine in cancer therapy. First, the multiscale modeling of complex mechanical microenvironments in humans and animals remains a formidable task. Researchers often study a single type of mechanical stimuli on cancer cells *in vitro*, while these stimuli are often combined *in vivo* to modulate cellular and molecular events. Replicating multiple types of mechanical stimuli *in vitro* is essential for comprehending cellular responses and understanding cancer development from a global perspective. Second, there is an attractive prospect of integrating quantitative data obtained from multiscale biomechanics measurements and predictions to create a virtual mechanical tumor. This integration involves several steps such as data pre-processing, feature proposal and model training, which may vary depending on the virtual mechanical organ/tissue and the type of inspection data. The development of state-of-the-art biomechanical experimental techniques and computer-based simulation approaches or even artificial intelligence algorithms will provide further information with high spatiotemporal resolution strategies, offering insights into molecular and cellular mechanisms from the perspective of mechanobiology or mechanomedicine. Third, numerous cancer mechanotherapy strategies target nuclear mechanics (e.g. high-frequency LIPU therapy, shock-wave therapy) or mechanotransduction (e.g. mechanical stretch therapy, low-frequency LIPU therapy) due to the mismatched mechanophenotyping between cancer cells and normal cells. However, there are still many problems to be solved. For example, the synergistic participation of mechanical signals and biochemical signals in cancer mechanotherapy remains unclear. It is unclear whether commonalities exist among different cancer species in mechanical signals mediating tumor cell apoptosis and the mechanisms of chemoradiotherapy resistance. With the progress of cutting-edge technologies such as three-dimensional organoid models, laser capture microcutting, single-cell multiomics sequencing and artificial intelligence, it is possible to identify the characteristics of different local microenvironments within the same cancer species and the common characteristics of microenvironments among different cancer species. This is expected to bring breakthrough changes to the reversal of tumor progression and chemoradiotherapy resistance in clinical tumors. Alongside conventional cancer treatments, mechanotherapy-based therapeutic systems will be developed for use in clinics.

## Conclusions

In conclusion, this review emphasizes the significance of mechanical cues in tumor cell apoptosis, including both extracellular force and intracellular force. Throughout the cancer development process, alterations in the extracellular mechanical microenvironment elevate the risk of malignancy, foster tumor progression and induce tumor cell phenotypes resembling stem cells to exacerbate tumor aggression and concurrently hinder drug delivery and immunotherapy. On the other hand, intracellular force can modulate mechanotransduction and intracellular force transmission, initiating a positive feedback loop that further shapes mechanical microenvironment and facilitates carcinogenesis. Clearly, mechanical cue has a critical role in regulating tumor development and treatment. Therefore, the application of the mechanomedicine concept in cancer holds the promise of designing scaffolds and delivering drugs for more effective therapeutic interventions.
